# Buffering Adaptive Immunity by Hydrogen Sulfide

**DOI:** 10.3390/cells11030325

**Published:** 2022-01-19

**Authors:** Giulia Pozzi, Giuliana Gobbi, Elena Masselli, Cecilia Carubbi, Valentina Presta, Luca Ambrosini, Marco Vitale, Prisco Mirandola

**Affiliations:** 1 Anatomy Unit, Department of Medicine and Surgery, University of Parma, Via Gramsci 14, 43126 Parma, Italy; giulia.pozzi@unipr.it (G.P.); giuliana.gobbi@unipr.it (G.G.); cecilia.carubbi@unipr.it (C.C.); valentina.presta@unipr.it (V.P.); luca.ambrosini@unipr.it (L.A.); marco.vitale@unipr.it (M.V.); 2 University Hospital of Parma, AOU-PR, Via Gramsci 14, 43126 Parma, Italy; 3 Italian Foundation for the Research in Balneology, Via Po 22, 00198 Rome, Italy

**Keywords:** gasotransmitters, NaHS, cystathionine-synthase, cystathionine-lyase, sulphurous waters

## Abstract

T cell-mediated adaptive immunity is designed to respond to non-self antigens and pathogens through the activation and proliferation of various T cell populations. T helper 1 (Th1), Th2, Th17 and Treg cells finely orchestrate cellular responses through a plethora of paracrine and autocrine stimuli that include cytokines, autacoids, and hormones. Hydrogen sulfide (H_2_S) is one of these mediators able to induce/inhibit immunological responses, playing a role in inflammatory and autoimmune diseases, neurological disorders, asthma, acute pancreatitis, and sepsis. Both endogenous and exogenous H_2_S modulate numerous important cell signaling pathways. In monocytes, polymorphonuclear, and T cells H_2_S impacts on activation, survival, proliferation, polarization, adhesion pathways, and modulates cytokine production and sensitivity to chemokines. Here, we offer a comprehensive review on the role of H_2_S as a natural buffer able to maintain over time a functional balance between Th1, Th2, Th17 and Treg immunological responses.

## 1. Introduction

T lymphocytes develop from CD7^+^CD34^+^ lymphoid progenitors, generated in the bone marrow and differentiated in the thymus. During thymic selection, they develop the ability to discriminate between self and non-self. T lymphocytes can be grouped into two main categories: helper CD4^+^ T cells, that regulate the whole immune response, and cytotoxic CD8+ T cells, that actively kill pathogens. Since T cells are essential components of adaptive immune responses, impaired T cell functions ultimately lead to immunodeficiency, promoting pathogen infections as well as various forms of tumors. Autoimmune disorders caused by uncontrolled autoreactive T cells include multiple sclerosis, rheumatoid arthritis, inflammatory bowel disease, diabetes, psoriasis, and autoimmune thyroiditis [1,2,3].

**Figure 1:**
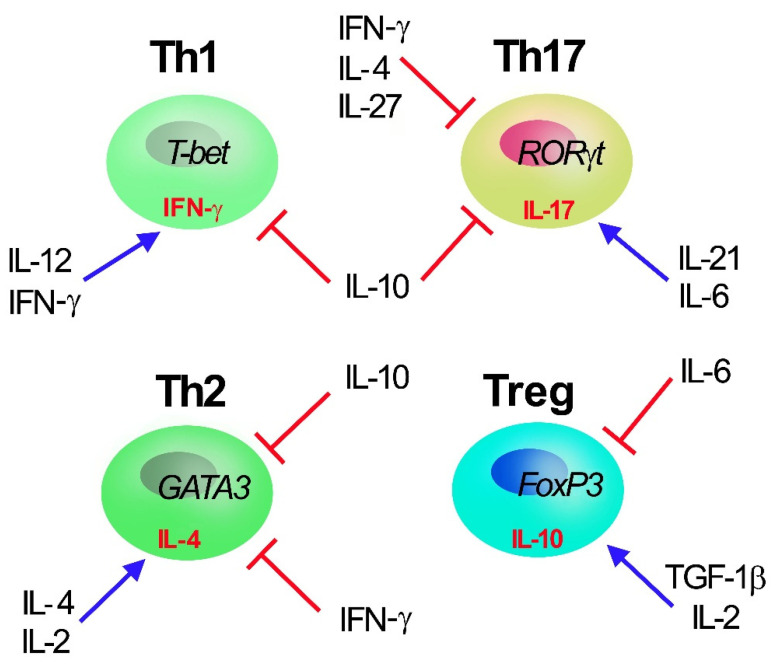
Th1, Th2, Th17, and Treg T CD4+ subset cells. Master transcription factors promoting Th polarization are reported inside cells (T-bet, GATA3, RORγT and Foxp3 for Th1, Th2, Th17, and Treg cell, respectively) together with selective secreted cytokines (γ-IFN, IL-4, IL-17 and IL-10 for Th1, Th2, Th17, and Treg cell, respectively). The main cytokines (IL-2, IL-4, IL-6, IL-12, IL-10, IL-21, IFN-γ, and TGF-1β) regulating Th polarization are reported: IL-10, secreted by Treg, acts as major inhibiting factors of Th polarization and proliferation.

T-helper (Th) cells have key functions in adaptive immunity and are involved in autoimmunity, asthma, allergy reactions, and tumor immunity. During T cell receptor (TCR)-mediated activation in the presence of specific cytokines in the surrounding microenvironment, naïve CD4^+^ T cells can polarize into one of multiple Th cell lineages, including Th1, Th2, Th17, and regulatory T (Treg) cells (Figure 1). Differentiation of different CD4^+^ effector/regulatory T-cell subpopulations is predominantly induced by specific sets of cytokines and finely tuned by different signaling pathways and transcription factors [4,5,6,7]. Th1 cells produce interferon-γ (IFN-γ), boosting cell-mediated immunity towards intracellular infections, whereas Th2 cells release interleukin (IL)-4, promoting humoral immunity to parasitic helminths. Th17 cells produce IL-17 and may have adapted to defend humans against microorganisms that Th1 and Th2 responses are not specific for, such as invasive bacteria as well as certain fungi [8,9,10]. The peculiar characteristic of IL-17 is that it has a potent activity on stromal cells in all tissues, leading to the production of inflammatory cytokines and chemiotaxis of leukocytes, particularly neutrophils, thus linking innate to adaptive immunity. Despite their significant role in host defense, Th17 have attracted great interest in recent years for their contribution in the pathogenesis of several autoimmune and inflammatory diseases [11]. Indeed, Th17 are pro-inflammatory T cells, and when in excess they promote autoimmunity and tissue damage. On the other hand, Treg cells, characterized by the expression of forkhead box transcription factor FoxP3, are required for immunological self-tolerance and homeostasis. They inhibit a wide range of immune responses (activated by Th1, Th2, and Th17 cells) as well as undesired immunity against a multitude of antigens, such as self-antigens, bacteria-originated antigens, and exogenous allergens. As a result, a deficiency in Treg cell population can result in acute inflammatory disorders such as autoimmunity, colitis, and allergies [12,13]. 

Endogenous hydrogen sulfide (H_2_S) exerts a variety of physiologically relevant activities. It belongs to the “gasotransmitter” family, along with nitric oxide (NO), carbon monoxide (CO), and sulfur dioxide (SO_2_). Once considered as poisonous and possibly fatal gases, they are now recognized as crucial intracellular signaling molecules with a wide range of physiological activities, and several H_2_S-releasing compounds are currently in preclinical and clinical trial, showing promising effects and therapeutic potential [14]. Specifically, the relevance of H_2_S in immune and inflammatory responses has long been a relevant topic of scientific research. H_2_S has been shown to modulate several immune cell activities, including monocyte and polymorphonuclear cell apoptosis, leukocyte adhesion and infiltration, T-cell activation, proliferation, and inflammatory cytokine production. Autoimmune disorders, neurodegenerative diseases, asthma, acute pancreatitis, and sepsis have all been related to the impact of H_2_S in inflammation [15,16,17,18]. Interestingly, H_2_S has been demonstrated to modulate T-cell lineage polarization, therefore representing a new and potential target to modulate and improve adaptive immunity responses.

## 2. Hydrogen Sulfide Biology, Intracellular Signal Transduction and Potential Targets

### 2.1. Hydrogen Sulfide Biology

**Figure 2:**
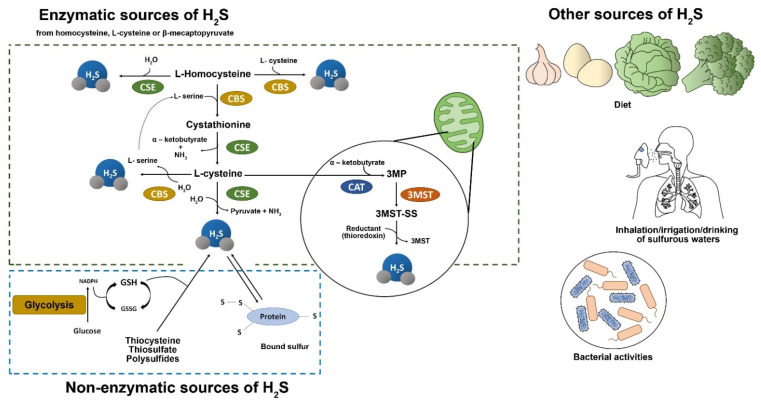
Source of H_2_S. There are four enzymatic pathways (dotted green rectangle) for the biosynthesis of H_2_S, including CBS, CSE, 3MST coupled with CAT, and 3MST that utilize L-cysteine, L-Homocysteine, and 3-mercaptopyruvate (3MP) as substrates. CBS and CSE may generate H_2_S in the cytosol whereas 3MST mainly resides and synthesizes H_2_S in mitochondria. A small portion of endogenous H_2_S is derived via nonenzymatic reduction (dotted blue rectangle). In the presence of reducing equivalents such as NADPH and NADH, reactive sulfur species in persulfides, thiosulfate, and polysulfides are reduced into H_2_S and other metabolites. Other sources of H2S are represented by diet, bacterial activities, inhalation, irrigation, and drinking of sulfurous waters. 3MP, 3-mercaptopyruvate; 3MST, 3-mercaptopyruvate sulfurtransferase; CAT, cysteine aminotransferase; CBS, cystathionine β-synthase; CSE, cystathionine γ-lyase; H_2_S, hydrogen sulfide; NADH, nicotinamide adenine dinucleotide; NADPH, nicotinamide adenine dinucleotide phosphate.

H_2_S was considered a lethal gas due to its flammability and corrosive properties but, more recently, it has been identified as a gaseous second messenger, alongside nitric oxide, and carbon monoxide [19]. H_2_S is soluble in both water and physiological fluids, it readily passes from water to air, and it volatilizes and is converted in the lungs in the presence of high oxygen concentrations [20]. Thus, H_2_S refers to a mixture of H_2_S, hydrosulfide and other sulfide species [19,21,22]. It is a colorless gas originating from geothermal activity, and it is found in plants as well as in synthetic compounds such as NaHS and GYY4137 [15,21,23,24,25,26,27]. Endogenous H_2_S is mainly synthesized from L-cysteine by cytoplasmic and mitochondrial cystathionine-synthase (CBS) and cystathionine-lyase (CSE) enzymatic activities, and is primarily generated by epithelial, vascular, and smooth muscle cells [23,28]. In addition, the combined activity of cysteine aminotransferase (CAT) and 3-mercaptopyruvate sulfurtransferase (3-MST) produces endogenous H_2_S in cytoplasm and mitochondria, respectively [15,21,23,29]. Moreover, non-enzymatic sources of H_2_S include glucose (through glycolysis), glutathione (GSH), inorganic and organic polysulfides, and bacterial activity in the gastrointestinal and respiratory mucosa [22,24,26] (Figure 2). H_2_S can directly act on its biological targets or be stored and metabolized. Finally, it is excreted by the kidneys through urine, intestine via flatus and lungs through exhaled air [23,28]. After synthesis, given its propensity to easily diffuse through lipid membranes without using specific transporters, H_2_S rapidly acts on its molecular targets expressed by several cells, including those in the respiratory, cardiovascular, and neurological systems, regulating several cellular processes [19,21,28,30,31,32,33]. The concentration of H_2_S is crucial in determining its biological functions in a variety of disorders. However, data on H_2_S concentration in plasma and extracellular matrix are extremely variable. Although several attempts to measure the plasma levels of H_2_S have been made, most of them resulted unfruitfully [34]. Many reviews usually mention baseline sulfide levels in plasma ranging from 1–100 μM, however these values could be biased by the chemical experimental conditions associated with the methods used. Therefore, the exact free and bioavailable sulfide concentration in blood and tissues is probably lower [19,34,35,36,37]. The substantial differences in the absolute values of baseline endogenous H_2_S levels reflect the differences in the analytical methods used by various groups. In plasma, H_2_S exists as a mix of approximately 20% H_2_S, 80% HS^−^ ion and a very low percentage of S^2−^ at a pH of 7.4 [37]. Moreover, the composition of sulfide forms in plasma is sensitive to temperature and pH, which affect the conversion of free form and bounded form as sulfates, sulfide, sulfonates, and elementary sulfur [36,37]. H_2_S plasma levels are also influenced by the interaction with blood cells as erythrocytes and plasma proteins [37,38,39,40]. Various methods have been developed to detect the amount of free sulfide and bound sulfide, obviously only when it is released from its bounded form. The proposed methods include colorimetric methods (such as direct or indirect methylene blue assay) [41], absorbance-based techniques [42], microfluids methods [43], gas and liquid chromatography [44,45], and electrochemical methods using ion-selective electrodes [46] and fluorescent probes [47]. Some of these methods induce protein desulfuration, thus affect the actual H_2_S measurement artificially elevating sulfide values (for instance, methylene blue assay). However, these methods have poor reliability and sensitivity, showing several limitations that are associated with the chemical characteristics of H_2_S, such as the propensity to permeate across cellular membranes, exceptionally short half-life rapid oxidation, rapid oxidation, and elevated reactivity with biological targets [48,49]. 

### 2.2. Hydrogen Sulfide Intracellular Signal Transduction Pathways

H_2_S can use a variety of signal transduction pathways for tuning its activities on specific tissues and organs. H_2_S can modify the activity of several kinases, including p38 mitogen-activated protein kinase (MAPK), extracellular signal–regulated kinase (ERK), and Akt signaling, by inhibiting or activating NF-κB nuclear translocation, resulting in a variety of cellular responses such as proliferation, cell death, differentiation, and cell cycle regulation. Indeed, H_2_S: (a) causes apoptosis stimulating ERK in human smooth muscle cells [50] and P38-MAPK in pancreatic cells [51]; (b) impacts the survival of human polymorphonuclear cells [52]; (c) inhibits IL-8 secretion by IL-21/IL-23 stimulated human keratinocytes [50,53,54]; (d) stimulates angiogenesis and vascular remodeling via the PI3K/Akt/survivin pathway in vascular smooth muscle cells [55]; (e) blocks the nuclear translocation of NF-κB, inhibiting a multitude of pro-inflammatory genes implicated in heart ischemic/reperfusion damage [56]. Administration of GYY4137 to rats results in potent anti-inflammatory effects through the decrease of the LPS-mediated upregulation of liver transcription factors NF-κB and STAT-3 [57]. Furthermore, H_2_S increases the nuclear localization of Nrf2 (a transcription factor that regulates the gene expression of several antioxidants) and the phosphorylation of protein kinase Cε and STAT-3 in an in vivo model of pharmacological preconditioning [58].

Cell signaling induced by H_2_S is otherwise necessary for mesenchymal stem cell (MSC) proliferation and differentiation. In fact: (a) PKC/Erk-mediated Wnt/β-catenin are required for bone differentiation [59,60]; (b) H_2_S decreases hypoxia-induced MSC apoptosis via PI3K/Akt, Erk1/2, and GSK-3β pathways [61,62,63,64]. Protein sulfhydration—that probably has a role in inflammation and endoplasmic reticulum stress [65,66]—occurs when H_2_S transforms cysteine residue -SH groups in specific proteins to hydropersulfide (-SSH), thus boosting their activity [16,65]. Sulfhydration has been described in GAPDH [67], K_ATP_ channels [65], p65 subunit of NF-κB [68], TRP calcium channel [59], and NFYB protein [69] activation. It has been shown that sulfhydration of the p65 subunit of NF-κB promotes macrophage survival, while a reduced sulfhydration of NF-κB promises interesting applications in tumors [66,68]. A considerable scientific effort has currently been made to understand the role of ion channels (K^+^, Cl^−^, and Ca^2+^) in H_2_S-dependent signaling and in the regulatory processes that govern it [70]. H_2_S exerts its protective effects against ischemia injury, hypertension, and apoptosis modulating inflammation, pain, and cell death by engaging K_ATP_ channels [16]. Cl^−^ channel, a cystic fibrosis transmembrane conductance modulator, has been implicated in H_2_S-mediated cell defense against oxidative stress in neuronal cells [71]. Moreover, evidence reveals that H_2_S targets L- and T-type Ca^2+^ channels, as well as TRP channels, for cardioprotection and inflammatory nociception [72], and excitatory signaling in cholinergic neurons, thus inducing neurosecretion [73,74,75].

The biosynthetic pathway, that supports H_2_S production via CSE, has been also involved in histone modifications, suggesting a role for H_2_S in epigenetically modulating inflammatory responses. Indeed, CSE knockout mice had higher levels of histone demethylase JMJD3 and lower levels of H3K27 methylation, while secreting higher levels of inflammatory cytokines IL-6 and IL-1. CSE has potent anti-inflammatory effects in rheumatoid arthritis through inhibition of JMJD3 expression by modulating the transcription factor Sp-1 [75]. Moreover, exogenous H_2_S decreased production of pro-inflammatory cytokines in an in vitro cell model, inhibiting histone acetylation and leading to chromatin remodeling [76,77]. However, even if histone acetylation and deacetylation alter chromatin remodeling during T cell growth and differentiation, there is still little information on HDAC and H_2_S in T cell functionality [78].

Currently, the pleiotropic activities of H_2_S, which apparently lack a common thread, suggest that H_2_S should rather be viewed in terms of system biology as a complex modulator of many molecular targets and their interactions.

## 3. H_2_S in T Cells

H_2_S-induced signaling plays an important functional role in T cell activation and polarization [28,56,79]. Accordingly, CBS, CSE and 3-MST are all expressed, although differentially, in T cell subsets and in naïve *versus* memory CD8^+^ T cells [56,80]. As observed during T cell activation, CSE and CBS expression are increased in polarized T cells as compared to naïve T cells, in which they seem virtually absent [81].

The effects of the exogenous H_2_S on T cell population appear to be closely related to the concentration range used in in vitro and in vivo experiments.

Indeed, exogenous hydrogen sulfide, administered at high concentrations (millimolar) causes caspase-independent/glutathione-dependent cell death in peripheral blood lymphocytes (CD8+ T cells and NK cells). Surviving lymphocytes showed dramatically reduced proliferation in response to mitogens and lower IL-2 production after 24 h of exposure to H_2_S. These findings show that H_2_S inhibits the cellular cytotoxic response and IL-2 production of peripheral blood lymphocytes, thus weakening primary players of local inflammatory reactions [82]. On the contrary, when H_2_S is administered at low concentrations (nanomolar/low micromolar), it increases T cell activation, and IL-2 production in mice [56]. T cell activation and proliferation are significantly inhibited when CBS or CSE expression are suppressed by siRNA, but restored by exogenous H_2_S. Hydrogen sulfide also increases the capacity of T cells to create immunological synapses by reorienting the microtubule organizing center (MTOC) and promoting tubulin-dependent cell polarization [56]. In summary, H_2_S promotes activation and proliferation of T cells with a characteristic bell-shaped dose-response curve, with a maximum positive effect at nanomolar concentrations and a toxic activity at higher concentrations (millimolar) [56]. This effect has been observed also in pathological conditions. Indeed, elevated concentrations of H_2_S inhibit excessive activation and proliferation of lymphocytes in lupus erythematosus patients [83]. Mechanistically, in activated T cells, CBS and CSE enzymes are inhibited by thrombospondin-1 (TSP1) that, via CD47 binding, reduces MEK-dependent ERK signaling thus counteracting the stimulatory effect of exogenous H_2_S donors [83].

Both innate and adaptive immunity rely on NF-κB [84]. It is known that H_2_S has a pro-inflammatory role in sepsis, mediated by NF-κB activation and subsequent elevation of transcription of NF-κB-dependent pro-inflammatory genes (IL-1, IL-6, TNF-α, MCP-1, and MIP-2) [85]. On the contrary H_2_S can reverse cell senescence and the pro-inflammatory impact of oxidative stress by boosting GSSH synthesis. Specifically, H_2_S causes the dissociation of nuclear erythroid factor 2-related factor 2 (Nrf2) and Kelch-like ECH-associated protein 1 (Keap1) via sulfhydration of Keap 1 at the Cys-151 residue and the formation of a disulfide bond between Cys-288 and Cys-613 residues, allowing Nrf2 nuclear translocation and binding to AREs [86,87]. However, little is known on the role of H_2_S-mediated activation of NF-κB or Nrf2 in T cells. In a lymphoblastic T cell line (CEM cell line), the administration of NaHS induced a significant down-modulation of NF-κB and HIF-1α expression, preventing their activities, and thus abrogating the downstream T cell adenosinergic signaling following hypoxia induction [88,89,90,91]. Since hypoxia has immunosuppressive effects in tumors [92,93,94], these data suggest that H_2_S administration might have beneficial effects in cancer, protecting T cell from hypoxia. Accordingly, it has been reported that the H_2_S-releasing compound diallyl trisulfide (DATS) significantly increased CD8^+^ T cells in mice models of melanoma, thus reducing the immunosuppressive activity of myeloid-derived suppressor cells [95]. In addition, H_2_S produced by sulfate-reducing bacteria increased the number of CD8^+^ T cells and the Th17 response in the mesenteric lymph nodes of a colitis mouse model, as described below [96].

The members of the human protein tyrosine phosphatases (PTP) family, known to be able to interfere with T cell signaling, are classified based on their structural and biochemical characteristics. PTP22, PTPN2, PTPN11, DUSP2, and DUSP6 have been shown to influence T cell subsets proliferation and function in an inflammatory bowel disease model [97,98,99]. Furthermore, it has been shown that various subsets of Th and Treg cells express varying quantities of the PTP enzyme [100]. The majority of PTP show a conserved catalytic domain that comprises a cysteine residue able to nucleophilic attack on a substrate. In some isoforms, like PTP1B, this catalytic residue can also be sulfhydrated. Although H_2_S can reversibly inactivate PTP1B, no data are available on the capacity of H_2_S to alter T cell polarization, proliferation, or evidence of crosstalk with PTP signaling via PTP1B [101].

H_2_S acts as an autocrine or paracrine enhancer of T cell activation when generated by activated T cells or when supplied exogenously (in the proper concentration range). However, it should be noted that, at higher doses, H_2_S decreases T cell survival and function. Therefore, to better clarify this topic, recent studies based on CBS or CSE knockout mice have explored the impact of H_2_S on T-cell activation and differentiation.

In ovalbumin (OVA)-induced acute asthma murine model, CSE knockout mice showed a worsening in allergen-induced airway hyperresponsiveness and developed acute asthma with a severe airway inflammation, characterized by Th2-mediated immune response cytokines. NaHS administration relieved asthma-related symptoms in CSE knockout mice, and reduced cell infiltrates and the levels of IL-5, IL-13, and eotaxin-1 in bronchoalveolar lavage fluid (BALF), indicating that H_2_S mediates a crucial protective role in the development of airway inflammation [102]. These data suggest that H_2_S might be a negative regulator of Th2-cell response.

Under baseline conditions, CSE knockout mice show no significant differences in CD4+ T cells, while presenting an increase of CD8^+^ T cells and of IFN-γ-releasing Th1 cells. During Mycobacterium tuberculosis (Mtb) infection, CSE knockout mice show a stronger adaptive immune response increasing the number of Th1 cells, decreasing neutrophils, and controlling Mtb growth in vivo [103]. Accordingly, it has been previously demonstrated that even if in CBS knockout mice their CD4+ T cell number did not changed, they presented an increase of IFN-γ and IL-17 (but not IL-4) producing CD4+ T cells. Moreover, when Treg cells were polarized to Th1, Th2 or Th17, CBS knockout Treg cells promoted Th1 and Th17, but not Th2, differentiation [69]. It has been reported that CSE can control Th1 responses, leading to immunological tolerance also in case of transplantation, although H_2_S was not considered the main mediator [104]. In chicken models, the capacity of H_2_S to balance Th1 vs Th2 responses has been investigated. Several cytokines (IL-1, IL-4, IL-6, and TNF-α) were upregulated by H_2_S inhalation, however IFN-γ was dramatically down-regulated. H_2_S activity was detected both in untreated animals and in the presence of LPS, although it was stronger in the presence of immunostimulants [105,106].

Similarly, H_2_S administration was reported to have positive effects in a model of bleomycin-induced lung fibrosis: these responses were related to an increase in IL-4 production and a decrease in IFN-γ expression, indicating a shift towards Th2 response [107]. The ability of the innate immune system to impact the adaptive immune response is widely established.

Similarly, the soluble components of the tumor micro-environment and the cellular elements (endothelial cells, mesenchymal stromal cells, Treg, antigen presenting cells (APC), dendritic cells, myeloid-derived suppressor cells, natural killer lymphocytes) are required for T cell immune response [108]. Since H_2_S appears to influence the oncogenic and immunogenic features of tumor cells, as well as various classical and non-canonical oncogenic signaling pathways [109,110], we checked for any data on the effects of H_2_S on the crosstalk between T cells and innate immunity in cancer patients.

Inhibition of endogenous H_2_S generation has recently been shown to boost the expression of activating/co-stimulatory ligands on breast cancer cells and improve their sensitivity to NK cell- and T cell-mediated immune responses [111]. Youness at al. demonstrated that endogenous H_2_S primarily mediates its effects via the miR-155/NOS_2_/NO axis. H_2_S suppresses the production of the NKG2D ligands MICA and ULBP2, reducing NK cell cytotoxicity against H_2_S-producing tumor sites. Furthermore, H_2_S inhibits the killing activity of chimeric antigen receptor transduction (CAR) T cells. This seems to be mediated in part by the downregulation of co-stimulatory ligands (CD86 and 4-1BB ligand) in H_2_S-producing tumor cells, which limits activation of cognate receptors on CAR T cells [111]. These findings pave the way for proteome analyses in in vivo administered H_2_S animal models [112]. H_2_S has been shown to activate a wide range of metabolic pathways that lead to lung injury in pigs, resulting in a reduction in antigen presenting ability, increased activation of the complement system, and mucus accumulation, which may induce immune suppression and facilitate inflammation in the lungs [112].

Overall, although further research is needed in the field, these data suggest that H_2_S plays a role in the crosstalk between T cells and innate immunity during immunogenic reactions.

## 4. Role of H_2_S in Th17 Cells

Th17 cells have been widely investigated in various diseases, including inflammatory bowel disease (IBD), colorectal tumors, autoimmune arthritis, psoriasis, hypoxia-induced pulmonary hypertension, and ischemic brain injury (HBI) [112,113,114,115,116,117]. Altogether these studies demonstrate that Th17 cells exert a role in the pathogenesis of inflammatory diseases, while also having a beneficial role in maintaining health [118].

Physiologically, intestinal bacteria are required to maintain a Th17 response in the mucosa [119,120,121]. However, increased Th17 cells and related cytokines (such as IL-17, IL-21 and IL-22) are linked to inflammatory disease severity, such as in IBD patients [122]. The role of H_2_S in the context of innate immunity in the mucosa has been explored in a colitis mouse model. Interestingly, it has been demonstrated that sulfate-reducing bacteria (SRB), that produce H_2_S, potentiate the mucosal Th17 response [96]. Indeed, SRB colonization enhanced the number of CD11b+, B, and T cells and boosted the formation and/or activation of Th17 cells in the mucosal immune system, as confirmed by upregulation of IL-6 and IL-17 by mesenteric lymph node cells in germ-free mice. Accordingly, H_2_S was demonstrated to influence type 2 immunity being a potent inducer of pro-inflammatory Th17 cells and Tregs in the intestine [123].

The relative numbers of the three lymphocyte subsets Th1, Th2, and Th17 are imbalanced in HBI. Upon HBI T-cell activation shifted to a pro-inflammatory Th1 setting while having no effect on the Th17 response [124]. While it is known that H_2_S levels and its enzymes are dysregulated following HBI, it was only recently explored the hypothesis that they may influence immune cell functions in neonatal mice, including local microglia and infiltrating peripheral immune cells [125,126,127]. Increase of H_2_S levels was obtained using L-Cysteine, a common substrate for its production [14,128]. H_2_S treatment inhibited CD4+T cell infiltration while simultaneously dramatically lowering the fraction of Th1 cells and increasing the Th17/Th2 ratio following HBI. These results suggest that L-Cysteine exerts anti-inflammatory effects by increasing the shift of T cells to Th2 response [127]. It is not clear whether L-Cysteine modulates only the recruitment of Th subpopulations and/or Th polarization in the HBI context.

Th1 and Th17 cells can cooperate and promote the development of autoimmune diseases [129]. Indeed, psoriasis was once thought to be a Th1-mediated skin disorder, but the attention has recently switched to IL-17-producing cells, such as Th17 lymphocytes [130]. Interestingly, patients affected by psoriasis have significantly higher homocysteine (Hcy) level in serum which is responsible for the pathologic stimulation of Th1 and Th17 cells [131]. Under physiological conditions, Hcy is metabolized to cysteine, which then produces H_2_S. On the contrary, in pathological conditions, high levels of Hcy inhibit CSE activity and reduce endogenous H_2_S generation. Accordingly, certain H_2_S donors have been reported to suppress Hcy levels, limiting Th1 and Th17 overactivation in psoriasis [132,133].

Diet is a means to increase H_2_S bioavailability [134,135]. As an example, the main biologically active molecules of garlic are amino acids, vitamins, micronutrients, and organosulfur compounds (OSCs), the latter being able to raise endogenous H_2_S [135,136]. It has been shown that pretreatment with a mixture containing dipropyl polysulfides (DPPS), components of garlic [137], significantly mitigated Concanavalin A (ConA)-induced hepatitis in mice. DPPS pretreatment reduced inflammatory cytokines while increasing Treg lymphocytes in the livers of ConA mice. DPPS demonstrated hepatoprotective benefits in ConA-induced hepatitis, as evidenced by reduced inflammation and a shift in the Th17/Treg balance in favor of Treg cells, implying possible applications of DPPS mixtures in inflammatory immune-mediated liver disorders [138]. Furthermore, Diallyl Trisulfide (DATS), an organosulfur molecule isolated from garlic bulbs, reduced inflammatory cytokine production, and controlled immune function in a collagen-induced arthritis mouse model. The suppression of the NF-κB and Wnt signaling pathways restored the equilibrium between Th17 and Treg cells [139]. It is commonly acknowledged that an imbalance in Th17/Treg levels is deleterious to RA. Adjustment of these imbalances may reduce joint inflammation and improve disease prognosis, implying a role for DATS as anti-arthritic drugs.

## 5. Role of H_2_S in Treg

T regulatory cells, commonly known as Tregs, play an important role in immunological homeostasis and self-tolerance. The presence of CD4, CD25, and FoxP3, a critical transcription factor for Treg polarization, distinguishes naturally occurring Tregs (nTregs). A subgroup of Treg cells exists in parallel to nTregs, named induced Tregs, (iTregs). Both iTregs and nTregs regulate immunological activation in a number of ways, both directly and indirectly. The capacity to direct Treg activities might represent an innovative strategy to prevent/treat autoimmune diseases, improve transplant tolerance, and stimulate immune activity against tumors [140,141,142]. Tregs express high levels of CBS and 3-MST but have a low CSE expression [3,69]. Blocking CBS and CSE function in mice reduces the amount of FoxP3+ Tregs, indicating that these enzymes play a role in the T cell polarization and/or maintenance of Tregs [69]. CBS knockout mice have less Tregs, and the reduction of Tregs cells is linked to immune cell infiltration and higher autoantibody production in different anatomical sites. H_2_S signaling promotes Treg hypomethylation, a crucial aspect of Treg phenotype, by boosting the production of the ten-eleven translocation (Tet) molecules, which are engaged in functional DNA demethylation. The sulfhydration of NFYB (nuclear transcription factor Y subunit beta) was discovered to be crucial in this context and it occurs probably via CSE-originated H_2_S or polysulfide compounds [69]. In a mesenchymal stem cell (MSC)/T cell coculture model, the involvement of H_2_S in driving T cell polarization towards Treg cells and in inhibiting Th17 cell polarization, was also established in in vitro system [143]. MSCs stimulated T cell polarization to Tregs, but this activity was reduced when CBS was knocked down. Pharmacological H_2_S treatment, by NaHS administration, partially reversed this effect, indicating that H_2_S was essential to retain immunomodulatory activity of MSC [143]. In an elegant recent study on M. tuberculosis infection (Mtb), it has been reported that in the alveoli of CSE knockout mice the number of Treg cells increased after infection [103]. Specifically, four weeks after infection, Treg cells reached a higher level than wild type mice that, in turn, do not retain increased Treg cells and, as a result, do not show an excessive Treg-mediated immune-regulation. These data obtained in Mtb-infected wild type mice are consistent with previous ones showing that high levels of H_2_S limit the release of pro-inflammatory molecules, including IL-1, IL-6, TNF-α, NO, and mitochondrial-reactive oxygen intermediates, but promote the secretion of the anti-inflammatory cytokine IL-10 [144,145,146]. Accordingly, in a model of colitis, H_2_S is produced by SRB, which up-regulate Th17 and Treg cytokine profiles (IL-10 increase, IL-2 decrease) in T cells from the mesenteric lymph nodes [92].

**Figure 3:**
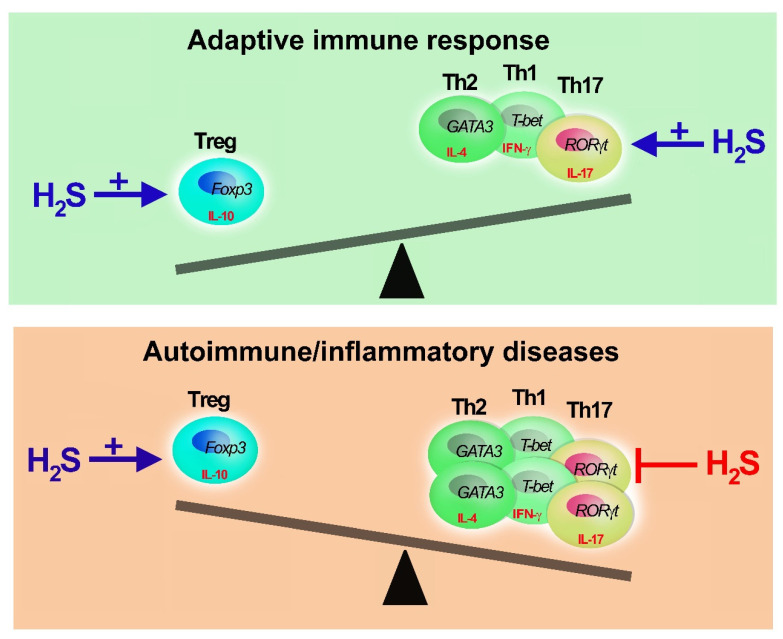
Adaptive immune response, H_2_S buffering activity. Hydrogen sulfide can restore the equilibrium of Th and Treg cells. H_2_S is needed to develop appropriate Th-mediated immune response promoting Th and Treg polarization and functions. In case of excessive Th1, Th2 or Th17 activation (unbalanced of immune response), as in immune-mediated diseases, H_2_S promotes Treg proliferation (+) and inhibits (−) Th activity and expansion. However, when H_2_S reaches millimolar doses, it has immunosuppressive activities impairing T cell proliferation and cytokine secretion.

Overall, while the evidence for a H_2_S role in Treg polarization is limited, it is suggested that this gaseous mediator plays an essential, non-redundant role in the modulation of adaptive immunity by stimulating Treg growth and activity (Figure 3).

## 6. Conclusions

Interestingly, a number of studies found that sulfur-containing and releasing compounds are important immunomodulators, particularly in the inflammatory T-dependent response, that typified immune-mediated diseases, such as ischemic brain injury, hepatitis, psoriasis, and arthritis. While H_2_S has long been known to play a role in modifying Th1/Th2 equilibrium, more recently, its effects on Th17, whose balance with Treg is crucial for adaptive immunity, have begun to emerge. However, further studies are needed to completely understand the role of H_2_S in the modulation of Th17/Treg responses, as well as how sulfur-containing substances play a part in this process.

**Figure 4:**
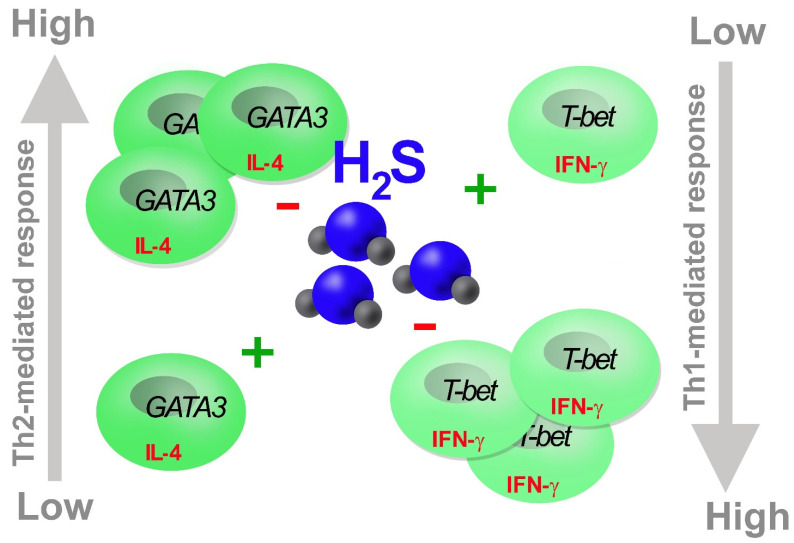
H_2_S balances Th1/Th2 response. H_2_S boosts (+) Th response limiting (−) excessive Th proliferation and activity obtaining an optimal balancing of Th1 and Th2 effects.

In this review, we have discussed the functional relevance of H_2_S as a T cell response buffer, blunting both positive and negative T cell response imbalances. Specifically, when a prompt Th response is required, it favors Th1 against Th2 response, coherently inhibiting Th17 and promoting Treg polarization, which limit the immune response. Accordingly, when T cell activity is dysregulated, like in Th1 and Th2-induced autoimmune disorders, exogenous H_2_S at physiological doses restores the Th response, rebalancing Th1 vs. Th2 subsets (Figure 4).

Therefore, slow-releasing H_2_S donors, activators of endogenous H_2_S-generating enzymes, and inhalation of sulfurous waters can be considered as long-term strategies to maintain over time a healthy balance between Th1 and Th2 immunological responses.

## Data Availability

Not applicable.
